# Discerning applicants’ interests in rural medicine: a textual analysis of admission essays

**DOI:** 10.3402/meo.v20.27081

**Published:** 2015-03-19

**Authors:** Carol L. Elam, Anthony D. Weaver, Elmer T. Whittler, Terry D. Stratton, Linda M. Asher, Kimberly L. Scott, Emery A. Wilson

**Affiliations:** 1 Office of Medical Education, University of Kentucky College of Medicine, Lexington, KY, USA; 2 Department of Internal Medicine, University of Kentucky College of Medicine, Lexington, KY, USA; 3 Center of Excellence in Rural Health, Hazard, KY, USA; 4 University of Kentucky College of Medicine, Lexington, KY, USA; 5 Office of Rural and Community Health, University of Kentucky College of Medicine, Lexington, KY, USA

**Keywords:** admissions, medical student selection, rural medicine, application essays, rural medical programs

## Abstract

**Background:**

Despite efforts to construct targeted medical school admission processes using applicant-level correlates of future practice location, accurately gauging applicants’ interests in rural medicine remains an imperfect science. This study explores the usefulness of textual analysis to identify rural-oriented themes and values underlying applicants’ open-ended responses to admission essays.

**Methods:**

The study population consisted of 75 applicants to the Rural Physician Leadership Program (RPLP) at the University of Kentucky College of Medicine. Using WordStat, a proprietary text analysis program, applicants’ American Medical College Application Service personal statement and an admission essay written at the time of interview were searched for predefined keywords and phrases reflecting rural medical values. From these text searches, derived scores were then examined relative to interviewers’ subjective ratings of applicants’ overall acceptability for admission to the RPLP program and likelihood of practicing in a rural area.

**Results:**

The two interviewer-assigned ratings of likelihood of rural practice and overall acceptability were significantly related. A statistically significant relationship was also found between the rural medical values scores and estimated likelihood of rural practice. However, there was no association between rural medical values scores and subjective ratings of applicant acceptability.

**Conclusions:**

That applicants’ rural values in admission essays were not related to interviewers’ overall acceptability ratings indicates that other factors played a role in the interviewers’ assessments of applicants’ acceptability for admission.

The maldistribution of physicians away from rural, often underserved areas represents a persistent and longstanding public health concern. Rural areas average only 5.3 primary care physicians (PCPs) and 5.4 specialists per 10,000 population, compared with 7.8 PCPs and 13.4 specialists per 10,000 population in urban areas (1). Furthermore, although roughly 20% of the US population lives in rural communities, only 11% of physicians graduating from medical school between 1988 and 1997 chose to practice in such locations (2). Problems of limited access to basic medical services in rural communities are further compounded by lagging economic and educational opportunities, high rates of chronic illness, and higher levels of uninsured and underinsured health coverage (3).

Numerous studies have shown that certain demographic characteristics, such as coming from a rural background and having specialty plans for family medicine, are among the two most powerful predictors of practicing in a rural area (4). Older students, those who have volunteered in a developing country, and those who do not have university-educated parents are also influential factors (5). Finally, research suggests that pursuing a medical education at a regional campus (6) and completing medical training in rural communities (7, 8) increase the likelihood of rural practice.

In the state of Kentucky, 79 of 120 counties, nearly all of them rural, are officially designated as primary care health professional shortage areas (HPSAs) by the Health Resources and Services Administration (HRSA) (9). To maintain current rates of utilization, Kentucky will need an additional 624 PCPs by 2030, a 24% increase compared to the state's 2010 PCP workforce (10). Clearly, Kentucky's medical schools must become adept at identifying students likely to practice in rural areas. Thus, to complement research on student characteristics at matriculation, as well as educational strategies to improve the rural physician workforce, this study examines student essays to identify potential ‘markers’ of rural medical interests that may exist at the admission stage.

## Rural Physician Leadership Program

In response, the University of Kentucky College of Medicine (UKCOM) established the Rural Physician Leadership Program (RPLP) in 2008 to attract applicants interested in practicing medicine in rural Kentucky. Up to 10 students admitted annually complete their preclinical (M1–M2) coursework at the University of Kentucky campus (Lexington) and their clinical (M3–M4) training at St. Claire Regional Medical Center in rural, central Appalachia (Morehead, KY).

To complement the established admission process at UKCOM (11), a separate but integrated admission protocol was developed for RPLP applicants based on input from Morehead's rural, community-based faculty. Using a nominal group process technique, faculty members were asked to list background characteristics and personal qualities that they deemed to be important for rural physicians to possess, and personal attitudes or skills that were critical to the success of RPLP students. Their responses were used to design the Morehead component of the RPLP selection process that includes an essay, rating scales, and a rural-oriented admission interview.

After a review of admission credentials, including the American Medical College Application Service (AMCAS) personal statement, selected applicants are invited to Morehead for an initial interview. There, applicants are given 20 min to write an essay in response to the prompt: *How does a physician in rural practice differ from a physician in urban practice?* Applicants are then interviewed by two rural health care providers to gauge their understanding of rural cultures and peoples, as well as their perceptions of rural physician-patient relationships. All interviews are open-file and include access to all student application materials.

The interviewers complete two rating forms. The first form summarizes applicants’ responses to the core topics explored during the interview, along with an overall ‘acceptability’ rating based on a seven-point Likert-type scale (ranging from ‘Unacceptable’ to ‘Outstanding, clearly superior applicant’); the second form asks interviewers to rate the consistency of applicants’ interests with those of rural practitioners. On this latter form, interviewers respond to the following prompts: ‘On a scale of 1–100 with 1 indicating no chance and 100 indicating absolute certainty, what is the likelihood that this applicant will establish practice in a rural Kentucky county?’; and ‘On a scale of 1–100, with 1 being not at all confident and 100 being completely confident, how confident are you in the accuracy of the above rating?’ These averaged ratings represent the ‘rural practice likelihood’ (RPL) score.

In launching this rural training track, we sought to explore rural medical values espoused by applicants in their AMCAS personal statements and rural essays. Because the AMCAS personal statement is written at the time of application and may be included in multiple applications sent to different medical schools, whereas the rural essay is program-specific and written at the time of interview, we wondered about the similarities and consistencies of rural medical values expressed in both narratives. We also wondered whether interviewers’ RPL scores or ‘acceptability’ ratings were associated with these expressed values. The purpose of this study, then, was to determine whether applicants’ expressed rural medical values, as assessed by predetermined words and phrases noted in their AMCAS and rural essays, were positively associated with perceived ‘likelihood’ of practicing in a rural community or ‘acceptability’ ratings.

## Methods

The study population was comprised of RPLP applicants who, over a 3-year period, were interviewed at the regional Morehead site (*n*=75). The following materials were examined for each applicant: 1) AMCAS personal statement; 2) rural essay; 3) overall interview rating for acceptability; and 4) RPL score. For each applicant, two rural-based interviewers independently assigned acceptability for admission to the program and RPL ratings. The two interviewer scores were combined for each scale, and a mean rating was calculated for each applicant.

WordStat and SimStat (12) were used to analyze narrative content and quantitative data, respectively. Referencing information collected in the earlier nominal group process exercise with Morehead clinical faculty, an initial lexicon of statements reflecting rural medical values was devised. Then the data dictionary of words connoting rural values was expanded using WordNet (13, 14), a large lexical dictionary of nouns, verbs, adjectives, and adverbs. Associated terms were grouped into sets of synonyms expressing a distinct concept (e.g., rural or rurality). By extending concepts such as distance, population density, amenities, and medical practice, the dictionary enabled the searching of phrases such as *rural physician, rural medicine, rural clinic, rural poverty and disparities*. Scores from 0 to 20 reflected the strength of rural medical values expressed in each essay.

After a Shapiro–Wilkes test indicated the data were not derived from a normal population, the nonparametric Kendall's tau b was selected to examine bivariate associations. The critical *p* value was specified @ ≤0.05, and all tests were one-tailed unless otherwise specified.

## Results

Not surprisingly, all 75 RPLP applicants expressed some rural medical values in their rural essays; however, nearly half (*n*=37) failed to do so in their AMCAS personal statement.

The mean number of statements reflecting rural medical values in the AMCAS personal statements and rural essays was 5.32 and 12.04, respectively. Examples of keywords and phrases from applicants’ essays that were indicative of rural medical values included desire to: ‘have a closer personal relationship with patients, become a community leader, return home to serve, treat the poor and those without insurance, and be personally recognized by patients and their family members’.

Interviewers’ average ratings of applicant acceptability ranged from 2.5 to 7.0 (SD =0.90), and did not differ significantly by site. Within respective pairs of interviewers, approximately three-quarters (74.1%) of acceptability ratings were perfectly concordant or within one rating point – which was identical by site. Interviewers’ average RPL scores ranged from 16 to 100% (SD=19.4%). However, rural interviewers’ RPL scores tended to be closer in agreement than their main-campus counterparts, and they tended to estimate applicants’ likelihood of rural practice slightly higher.

The rural medical values scores obtained from the AMCAS personal statement and rural essay were modestly but significantly related (*τ*_b_=0.25, *p*=0.007) indicating that RPLP applicants expressing rural medical practice values in their AMCAS personal statements expressed similar values in their rural essays. In turn, these rural medical values scores derived from the AMCAS (*t*_b_=0.26, *p*=0.005) and RPLP essays (*τ*_b_=0.23, *p*=0.004) were each similarly related to students’ assigned RPL scores. Finally, although interviewers’ ‘acceptability’ ratings were positively associated with RPL scores (*τ*_b_=0.26, *p*=0.002), they were not significantly related to applicants’ expressed rural medical values in either essay.

## Discussion

Despite being motivated to reduce health personnel shortages, the task of making admission decisions based on future predictions of specialty choice or practice location is both daunting and imperfect. Indeed, research has demonstrated the influence of many factors in trainees’ decisions about medical specialty and location of practice, including student characteristics, curricula, family relationships, the availability of jobs in various specialties, and accrued debt (15, 16). Given the complexity of this endeavor, admission committees often fail to recognize or appropriately weigh information used to make these important predictions (17).

As little has been written about the rationale, utility, or effectiveness of essay questions as a selection tool in general, and more specifically, how applicants approach essay questions (18), this study sought to explore whether applicants to our rural training track provided any textual ‘hints’ reflecting their interests or intentions. To that end, we used software to analyze AMCAS personal statements and rural essays for statements reflecting rural medical values – emphasizing their possible relationship to subjective ratings of acceptability for admission and likelihood to practice medicine in a rural setting.

Although all RPLP applicants made statements reflecting some rural medical values in their rural, program-specific essays, only about half did so in their AMCAS personal statements. As reported by White and colleagues, this discrepancy may arise from the tension between ‘expected responses’ of what applicants think will maximize their chances of acceptance and ‘genuine responses’ that allow applicants to ‘show themselves’ and ‘tell their own story’ (18, 19). In our case, the former may have dominated the rural essay, whereas the more general, less targeted AMCAS personal statement may have been more conducive to the latter. Although further qualitative analysis of the essays would be necessary to explore actual rural-related themes, the rural medical values score gleaned from both sources were significantly related to interviewers’ perceived likelihood of rural practice.

In general, RPLP interviewers are chosen based on their experience in rural medicine. However, although they are oriented on the use of the various admission ratings, how ratings should be assigned is not formally addressed. Instead, interviewers are free to assess applicants’ likelihood of rural practice vis-à-vis their own frameworks based on their professional judgment and impressions of the applicant. Thus, it remains unclear whether, in assigning a rating, a variable focus is made on oral or written comments, demographics, or other factors. These are important considerations in these and future results.

Several other limitations should also be noted. First, although our intent was to identify valid indicators of rural medical values, it is likely that our list was incomplete. Moreover, some references, such as ‘caring for the underserved’, may be equally applicable to urban, inner-city medical practice. Second, because of the relative newness of the program, we cannot yet report on students’ eventual practice locales. Finally, our focus on a novel program within a single institution necessarily limits the generalizability of these findings.

## Conclusions and implications

The RPLP is one school's attempt to address the persistent shortage of physicians in rural, often underserved communities. Subsequently, the goal is to select students with strong, ‘associated’ medical values in hopes of maximizing their chances of one day practicing in rural Kentucky. Textual analysis of admission essays appears to hold some promise as a means of exploring specific ideals, beliefs, or values of medical school applicants.

That the prevalence of rural medical values was unrelated to interviewers’ overall acceptability ratings suggests the role of other factor(s). Whether these are currently unspecified and unmeasured in our admissions process – or may be amenable to novel methodological approaches such as textual analysis of open-ended essays – remains a question for future research.
